# Combination of acquired von Willebrand syndrome (AVWS) and Glanzmann thrombasthenia in monoclonal gammopathy of uncertain significance (MGUS), a case report

**DOI:** 10.1186/s12959-018-0184-2

**Published:** 2018-11-26

**Authors:** Elizabeth Sarah Mayne, Malcolm Tait, Barry Frank Jacobson, Evashin Pillay, Susan J. Louw

**Affiliations:** Department of Molecular Medicine and Haematology, Faculty of Health Sciences, University of the Witwatersr and National Health Laboratory Services, 7 York Road, Parktown, Johannesburg, 2196 South Africa

**Keywords:** Acquired von Willebrand syndrome, Monoclonal Gammopathy, Acquired Glanzmann’s Thrombasthenia

## Abstract

**Background:**

Autoimmune paraphenomena, are associated with B-cell lymphoproliferative disorders, including monoclonal gammopathy of uncertain significance. These paraphenomena can rarely include acquired bleeding disorders.

**Case presentation:**

This case study reports an unusual clinical presentation of 2 acquired bleeding disorders, Acquired von Willebrand syndrome (disease) and Acquired Glanzmann’s thrombasthenia, in an elderly patient with monoclonal gammopathy of uncertain significance.

**Conclusions:**

Acquired bleeding disorders are often underdiagnosed and a high degree of clinical suspicion is required. The patient in this study demonstrated platelet aggregometry which was atypical for isolated Glanzmann’s thrombosthenia because of the severe concomitant endogenous decrease in von Willebrand factor. There was an absence of platelet aggregation to all tested agonists including ristocetin. Once the diagnosis was made, however, the patient showed a partial response to intravenous immunoglobulin confirming the immunological pathogenesis in this case. This case highlights the need to consider acquired bleeding disorders in patients with a possible predisposing factor.

## Background

Monoclonal gammopathy of undetermined significance (MGUS) is a pre-malignant B-cell condition characterized by the presence of a monoclonal protein and fewer than 10% clonal plasma cells in the bone marrow with no end-organ damage. MGUS is present in ~ 3% of the general population over 50 years of age with a 1% annual risk of progression to malignant myeloma (MM) [1]. MGUS is associated with an increase in auto-immune paraphenomena including haematological disorders. Autoimmune disorders on the other hand constitute a risk for the development of clonal plasma cells disorders [2].

Von Willebrand Factor (vWF) is a large multimeric glycoprotein which is essential in primary haemostasis, facilitating platelet adhesion at the site of vascular injury. It also increases the half-life of coagulation factor VIII by protecting it from proteolysis in the circulation [3]. Von Willebrand disease (vWD) is a primary genetic defect resulting in vWF dysfunction which may be mild or result in a significant bleeding diathesis [3]. Acquired von Willebrand syndrome (AVWS), a less prevalent disease, is a secondary structural or functional defect in vWF associated with a number of clinical conditions including autoimmune diseases and malignancies. Autoantibodies may interfere with vWF function or mediate increased clearance of the protein. Proteolysis may be associated with increased shear stress (for example, in cardiac valvular abnormalities). VWF may also be adsorbed onto transformed cells or platelets [4, 5].

Platelets play a fundamental role in primary haemostasis. Platelet abnormalities are often associated with mucocutaneous bleeding. Glanzmann’s thrombasthenia (GT) is a rare inherited platelet disorder caused by abnormalities in the GPIIb/IIIa (or αIIbβ3) receptor which binds fibrinogen, vWF, vitronectin and fibronectin to mediate platelet aggregation and clot retraction. Clinically, patients with GT present with a variable bleeding diathesis (most commonly epistaxis or menorrhagia) which may be severe requiring both repeated platelet and red cell transfusions. A similar clinical picture is seen in acquired Glanzmann’s thrombasthenia (AGT). Autoantibodies, again associated with various disease processes including malignancy, interfere with the function of the GPIIb/IIIa receptor [6].

Diagnosis of these acquired bleeding disorders can be challenging requiring high levels of clinical suspicion and specialized laboratory testing facilities. Treatment, in addition to management of the bleeding episodes, often requires immune modulation to suppress or remove autoantibodies.

## Case presentation

An 81-year-old female patient, Mrs. NM, presented to casualty with a 3-year history of melaena, haematemesis and lethargy. Previous medical history recorded 8 uneventful deliveries and multiple tooth extractions which were not associated with excessive bleeding. There was also no family history of pathological bleeding. On clinical examination, the patient had signs of cardiac failure but was haemodynamically stable. She had diffuse cutaneous ecchymoses and significant pallor. There was no clinical evidence of haemarthrosis. There was no organomegaly or lymphadenopathy and the neurological system was grossly intact. Laboratory testing revealed a severe normocytic, normochromic anaemia. Her renal function was normal. Her albumin was mildly reduced but liver enzyme levels were not elevated. Coagulation testing revealed a prolonged activated partial thromboplastin time and a mildly prolonged prothrombin time which both corrected on mixing studies (data not shown). The Factor VIII levels and von Willebrand Factor antigen and activity (Ristocetin co-factor) levels were markedly reduced. Platelet aggregation studies showed a markedly reduced response to all platelet agonists including high dose ristocetin. Platelet function analyser studies demonstrated prolonged closure to both collagen/epinephrine and collagen/ADP (Table 1).

**Table 1:** Summary of laboratory findings on NM at presentation and after each course of Intravenous Immunoglobulin demonstrating transient improvement

Laboratory parameter	Reference range	Presentation	First course IVIg	Second course IVIg	Third course IVIg
Full Blood Count					
Haemoglobin	12.4–16.7 g/dL	3.5	14.7	13.1	12.7
Haematocrit	0.35–0.49 UL	0.12	0.48	0.38	0.40
Red cell Count	3.8–5.5 × 10^9^/L	1.29	5.56	4.88	4.43
Mean Cell Volume	79–100 fL	89.90	85.60	85.60	89.40
Mean Cell Haemoglobin	27–35 pg	27.10	26.50	26.80	28.70
Mean Cell Haemoglobin concentration	29 - 37 g/dL	30.20	31.30	31.30	32.10
White Cell Count	4-12 × 10^9^/l	4.52	5.14	4.70	2.62
Platelet Count	150-450 × 10^9^/L	310	245	245	187
Red cell distribution width	11.0–16.0	22.80	14.10	14.10	13.20
Reticulocyte production					
Reticulocyte production index	1.00–1.25	2.2			0.9
Iron Studies					
Ferritin	20-300 ng/mL	7			11
Coagulation studies					
INR	1.00–1.25	1.06			1.02
Prothrombin Time	< 14 s	13.2		11.8	14.6
Activated Partial Thromboplastin Time	27–43 s	52.7			35.4
Fibrinogen	1.7–4.2 g/L	3.5			3.8
Factor VII levels					
Factor VIII	50–150%	6	48 (7^a^)	78 (15^a^)	88 (22^a^)
Factor VII inhibitor	Bethesda	0			0
Von Willebrand Factor Screen					
Von Willebrand Factor Activity	47.8–173.2%	15			32 (6^a^)
Von Willebrand Factor Antigen	50–160%	10	52 (10^a^)	51 (14^a^)	43 (10^a^)
Platelet studies					
Agonist - ristocetin 0.5μg/mL	No response	Normal		Normal	Normal
Agonist - ristocetin 1.5μg/mL	60–110%	5	10	10	32 (10^a^)
ADP 5.0 uM	60–100%	17			
Epinephrine 10μg/mL	60–110%	14			
Collagen 2μg/mL	60–110%	3			
Arachidonic Acid		Decreased			
Platelet function analyser					
Collagen/Epinephrine	82–150	> 300			
Collagen /Adenosine diphosphate	62–100	> 300			
Liver function tests					
Albumin	35–50 g/L	28			
Bilirubin (unconjugated)	3-6umol/L	4			
Bilirubin (conjugated)	2–7 umol/L	3			
AST	13–35 IU/L	8			
ALT	< 35 IU/L	3			
IgM paraprotein	g/L	3	4		4
Urea and Electrolytes					
Sodium	136-145 mmol/L	136			
Potassium	3.5–5.1 mmol/L	4.5			
Chloride	98-107 mmol/L	108			
Total CO_2_	21-29 mmol/L	23			
Urea	2.9–8.2 mmolL	2.5			
Creatinine	53–97 umol/L	36			
Bone Marrow Aspirate	Clonal plasma cells present but < 5% of cells. Iron stores absent.				

^a^Results prior to administration of intravenous immunoglobulin where appropriate

**Fig. 1:**
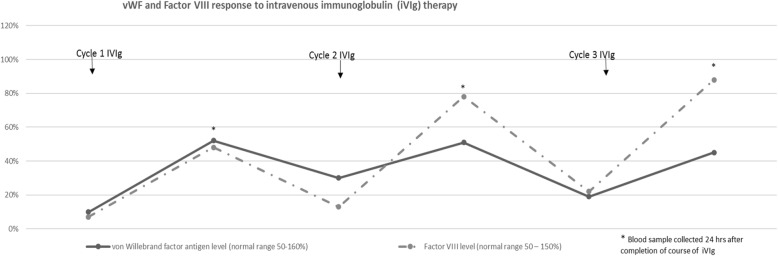
vWF and Factor VIII response to intravenous immunoglobulin (IVIg) therapy

Gastroscopy revealed mild duodenal angiodysplasia with active bleeding. The patient received multiple blood and coagulation factor transfusions, anti-fibrinolytic agents and proton-pump inhibitors to control the bleeding. Monthly cycles of intravenous immunoglobulin (Polygam) of 40 g daily for 3 days were commenced and 3 cycles were administered. This regimen resulted in a clinical response with cessation of the bleeding and partial transient recovery of the vWF and Factor VIII levels (Fig. 1). Ristocetin response on platelet aggregometry demonstrated partial improvement. Following clinical stabilization, the patient was discharged to outpatient follow-up and subsequently relocated to a rural treatment facility.

## Discussion and conclusions

Although the exact prevalence of AVWS is not known, it is a rare and potentially serious bleeding disorder with a multifactorial etiology [7]. AGT is equally rare requiring a high index of suspicion for timeous recognition [6]. This case study describes a patient who presented with both conditions in the background of an underlying MGUS. The response to intravenous immunoglobulin (Polygam) therapy suggests that an autoimmune paraphenomenon was responsible for the development of the acquired bleeding disorders.

The diagnosis of acquired bleeding disorders is often difficult given the heterogeneity of the clinical presentation. A detailed individual and family bleeding history, clinical examination and laboratory diagnostic tests are required [8]. In suspected AVWS, vWF antigen (vWF:Ag) and activity levels and factor VIII activity levels should be measured. In many cases, all 3 of these parameters are reduced [9]. A disproportionate reduction in activity:antigen ratio for vWF may indicate the presence of inhibitory antibodies or decreased high molecular weight (HMW) multimers. Although more specialized testing may refine the diagnosis and help to clarify the underlying mechanism of disease (including vWF multimer analysis, vWF collagen-binding (vWF:CB) assays and vWF pro-peptide analysis), these are not available in our centre [10]. vWF pro-eptide, which measure vWF biosynthesis, was previously suggested as a specific marker for AVWS diagnosis. An increased propeptide/vWF:Ag ratio, therefore, may represent an accelerated clearance of plasma vWF. An increased ratio is, however, also present in certain patients with congenital type I vWD mediated by accelerated vWF clearance. A sandwich enzyme-linked immunosorbent assay to detect and quantify anti-VWF antibodies has been described but lacks specificity [11]. The treatment of AVWS involves control of the bleeding episodes, prevention of bleeding related to elective invasive procedure and the control of the underlying disease [12].

AGT is characterized by decreased or absent platelet aggregation in the presence of adequate platelet numbers. The GPIIb/IIIa receptor levels are normal but receptors are dysfunctional because of inhibition by auto-antibodies which develop in a number of different conditions including haematological malignancies and autoimmune states [6]. The hallmark laboratory finding in GT is reduced platelet aggregation to all agonists (collagen, ADP, arachidonic acid and epinephrine) on light-transmission-aggregometry (LTA) with a normal response to ristocetin. Platelet functional analyser studies show prolonged closure values to Collagen/ADP and Collagen/Epinephrine. Ristocetin mediates vWF binding to platelet receptor glycoprotein Ib (GPIb) with subsequent platelet aggregation. The patient in our case study demonstrated reduced responses to all the platelet agonists including ristocetin which probably reflected the significantly decreased vWF levels related to the co-existing AVWD. Various therapies including corticosteroids, chemotherapeutic agents, plasma exchange, intravenous immunoglobulin, recombinant factor VIIa and rituximab as well as platelet transfusions have been employed with variable success in patients with AGT [13, 14].

Despite 30 years of accumulated experience in the diagnosis and management of AVWS and AGT, many aspects of these syndromes remain unclear and these disorders are rare. The possibility of acquired bleeding disorders should, however, be considered in the appropriate clinical setting and in patients with atypical laboratory features, multiple underlying mechanisms may be contributory [4, 6].

## Abbreviations

ADP: Adenosine diphosphate
AGT: Acquired Glanzmann thrombasthenia
AVWS: Acquired von Willebrand syndrome
GPIb: Glycoprotein Ib
GT: Glanzmann thrombasthenia
IVIg: Intravenous immunoglobulin
LTA: Light-transmission-aggregometry
MGUS: Monoclonal gammopathy of undetermined significance
MM: Multiple Myeloma
PFA-100®: Platelet function analyzer
vWD: Von Willebrand Disease
vWF: Von Willebrand Factor
